# Comparison of efficacy and safety between oral propranolol combined with and without intralesional injection of lauromacrogol for infantile hemangioma

**DOI:** 10.3389/fped.2024.1361105

**Published:** 2024-06-26

**Authors:** Fan Ma, Xiaoliang Liu, Chuan Wang, Hongyu Duan, Kaiyu Zhou, Fan Hu

**Affiliations:** ^1^ Department of Pediatric Cardiology, West China Second University Hospital, Sichuan University, Chengdu, Sichuan, China; ^2^ The Cardiac Development and Early Intervention Unit, West China Institute of Women and Children’s Health, West China Second University Hospital, Sichuan University, Chengdu, Sichuan, China; ^3^ Key Laboratory of Birth Defects and Related Diseases of Women and Children, Ministry of Education, Sichuan University, Chengdu, Sichuan, China; ^4^ Key Laboratory of Development and Diseases of Women and Children of Sichuan Province, West China Second University Hospital, Sichuan University, Chengdu, Sichuan, China

**Keywords:** infantile hemangioma, oral propranolol, lauromacrogol, combined treatment, efficacy and safety

## Abstract

**Aims and objectives:**

The purpose of this study was to compare efficacy and side effects between oral propranolol combined with and without intralesional injection of lauromacrogol for infantile hemangioma (IH).

**Material and methods:**

This was a single center randomized controlled prospective study, all participants were firstly diagnosed with IH between August 2022 and January 2023 in our hospital and without any treatment before. Patients were randomized into two groups. PRO group: oral propranolol (2 mg/kg/day) continued for 6 months; PRO + LAU group: oral propranolol (2 mg/kg/day) for 6 months and intralesional injection of lauromacrogol for 2–4 times within 6 months. The dimensions, color, consistency, photographic documentation were well recorded based on Visual Analogue Scale (VAS) before and after starting treatment. According to the treatment response after 6 months, the results were classified into four levels: Grade 1, complete resolution achieved; Grade 2, with ≥50% reduction in size of IH; Grade 3, with <50% reduction in size of IH; Grade 4, no response or worsening of IH.

**Results:**

A total of 67 patients were involved in the study (17 boys, 50 girls; mean age, 3.6 months, range, 1.1–7.2 months) and randomized to receive oral propranolol combined with or without intralesional injection of lauromacrogol (29 in PRO group, 38 in PRO + LAU group). All patients completed treatment. Eleven patients (37.9%) in PRO group were in Grade 1, 14 patients (48.3%) in Grade 2, 4 patients (13.8%) in Grade 3, compared with these in PRO + LAU group, 11 patients (28.9%) in Grade 1, 24 patients (63.2%) in Grade 2, and 3 patients (7.9%) in Grade 3. No patient was in Grade 4, and no severe side effects were observed in both group. In PRO group, it takes an average of 17.1 ± 5.4 weeks from the start of treatment to cure, and in PRO + LAU group, the average time is 13.7 ± 4.9 weeks.

**Conclusion:**

Oral propranolol with intralesional injection of lauromacrogol was a safety treatment strategy for IH. But it was not superior to oral propranolol in final cure rates (*P* = 0.45), moreover, it cannot certainly offer the benefits of shortening the duration of oral drug treatment (*P* = 0.24).

## Introduction

Infantile hemangioma (IH) is the most common vascular tumor in infancy, which attack approximately 4% children under 1-year-old (1–3). IH is usually diagnosed exactly within first few weeks of life, and it has a natural history involving three stages including rapid proliferative, stable and slow spontaneous degeneration stage (4). Some studies showed more than half of IH need to be treated because of incompletely regression, residual lesions, life-threatening location, or local complications (5).

Oral propranolol is the main treatment method now as its efficacy and minimal side effects (6, 7), however, a long-term follow-up study revealed that part of the IH patients treated with propranolol could still have serious residual lesions after regression or even without significant regression, which may need other additional treatment (8). Sclerotherapy is a traditional treatment method for IH in China through intralesional sclerosing agent by its strong antiangiogenic effect (9, 10). In recent years, intralesional injection of lauromacrogol, a new sclerosing agent, has been proven to be a safe and effective method for IH in many medical centers in China, with low incidence rate of swelling, ulceration, infection and pain (11, 12). Therefore, in this study we try to explore that whether intralesional injection of lauromacrogol can be a safe additional treatment for oral propranolol, and improve the effectiveness compare to use oral propranolol only.

## Material and methods

This was a single center randomized controlled clinical study conducted in West China Second University Hospital of Sichuan University between August 2022 and January 2023, and approved by Sichuan University Ethics Committee. Informed written consent was obtained from the parents after this study's nature was fully explained to them. All research was performed following the relevant guidelines and regulations.

For this study, the inclusion criteria were: (1) The age range in 2 weeks to 8 months; (2) Diagnosed as IH in our hospital firstly and without any treatment before; (3). The lesion located on the face, trunk, and limbs; (4) Without other diseases like: heart disease, cardiac arrhythmia, bronchoobstructive disease, diabetes mellitus, hypertension, hypotension. And only when the lesions had an impact on the appearance or health of the children, or when parents had a strong desire for treatment, the treatment was implemented. The exclusion criteria were: (1) Received any other treatment before; (2) The lesion was too thin to accept injection; (3) The lesion is located in the natural orifice or internal organs; (4) With any other disease mentioned before; (5) Accompanied by involvement of other organs or systems, like PHACE (an acronym for Posterior fossa brain malformations, segmental facial Hemangiomas, Arterial anomalies, Cardiac defects, Eye anomalies, and sternal clefting or supraumbilical raphe) or LUMBAR (Lower body hemangioma and other skin defects, Urogenital anomalies and Ulceration, Myelopathy, Bony deformities, Anorectal malformations and Arterial anomalies, and Renal anomalies).

The patients were randomized divided into 2 groups by the simple randomization. First group was propranolol (PRO) group: in this group, the patients received treatment by oral propranolol (2 mg/kg/day) only continued for 6 months; and in second group, propranolol + lauromacrogol (PRO + LAU) group, in addition to receiving oral propranolol (2 mg/kg/day) for 6 months, the patients also received intralesional injection of lauromacrogol for 2–4 times within 6 months. All patients were subjected to a detailed history and clinical examination, and initial cardiac evaluation.

All patients received reexamination every 4 weeks, and the dimensions, color, consistency were recorded, and photographs of the lesions were taken at the same time. Ultrasound was also used to evaluate changes in tumor bodies before and after treatment. Treatments were discontinued on complete resolution of IH or if intolerable side effects developed in this period. Clinical photographs were evaluated individually by 3 specialized doctors based on Visual Analogue Scale (VAS). According to the scoring system of Hesham et al. (13), treatment effectivity was divided into four grades based on the percentage of regression: Grade 1 (excellent final response), means complete resolution achieved; Grade 2 (good final response), means with ≥50% reduction in size of IH; Grade 3 (fair final response), means with <50% reduction in size of IH; Grade 4 (poor final response), no response or worsening of IH.

Statistical analysis was conducted with a statistical package (SPSS, version 18, IBM Corp, Armonk, NY). Data were summarized using mean, SD, and range for quantitative variables, and as number and percentages for categorical data. Normality of data was checked by measures of skewness and Kolmogorov-Smirnov test of normality. The Student *t*-test or Mann-Whitney test was used to compare mean values in the quantitative data analysis, while the chi-square test was used for qualitative data. *P*-values less than 0.05 were considered to be statistically significant.

## Results

Sixty-seven patients with IH were involved in this study, 17 of them were boys and 50 of them were girls, the age of them ranged from 1.1 to 7.2 months (mean age, 3.6 months). All patients were randomized to receive oral propranolol combined with or without intralesional injection of lauromacrogol. A total of 29 patients were in PRO group, and 38 patients were in PRO + LAU group. All patients completed the treatment and all information was well obtained and recorded in follow-up.

In total, single IH lesion was most common, 5 (7.4%) lesions were on the head, 17 (25.4%) lesions were on the face, 3 (4.5%) lesions were on the neck, 14 (20.9%) were on the limbs, 25 (37.3%) were on the trunk, and 3 (4.5%) of them were mixed IH. For the type of IH, 3 (4.5%) of them were superficial lesions, 42 (62.7%) of them were deep lesions and 22 (32.8%) were mixed lesions. The mean diameter of the lesions was 3.1 ± 1.9 cm in PRO group, and the mean diameter of the lesions was 2.7 ± 1.1 cm in PRO + LAU group. Information about baseline characteristics is shown in Table 1.

**Table 1:** Baseline characteristics of 67 patients and infantile hemangioma.

Item	PRO group	PRO + LAU group	*P*-value
Age, months (mean, SD)	3.9 ± 2.1	3.4 ± 1.9	0.28
Sex, male (*n*, %)	10 (34.5%)	7 (18.4%)	0.13
Lesion location (*n*)			
Head	2	3	0.65
Face	7	10	
Neck	1	2	
Limb	9	5	
Trunk	9	16	
Mixed	1	2	
Type of IH (*n*)			
Superficial	2	1	0.69
Deep	18	24	
Mixed	9	13	
Diameter of the lesion, cm (mean, SD)	3.1 ± 1.9	2.7 ± 1.1	0.24

**Figure 1:**
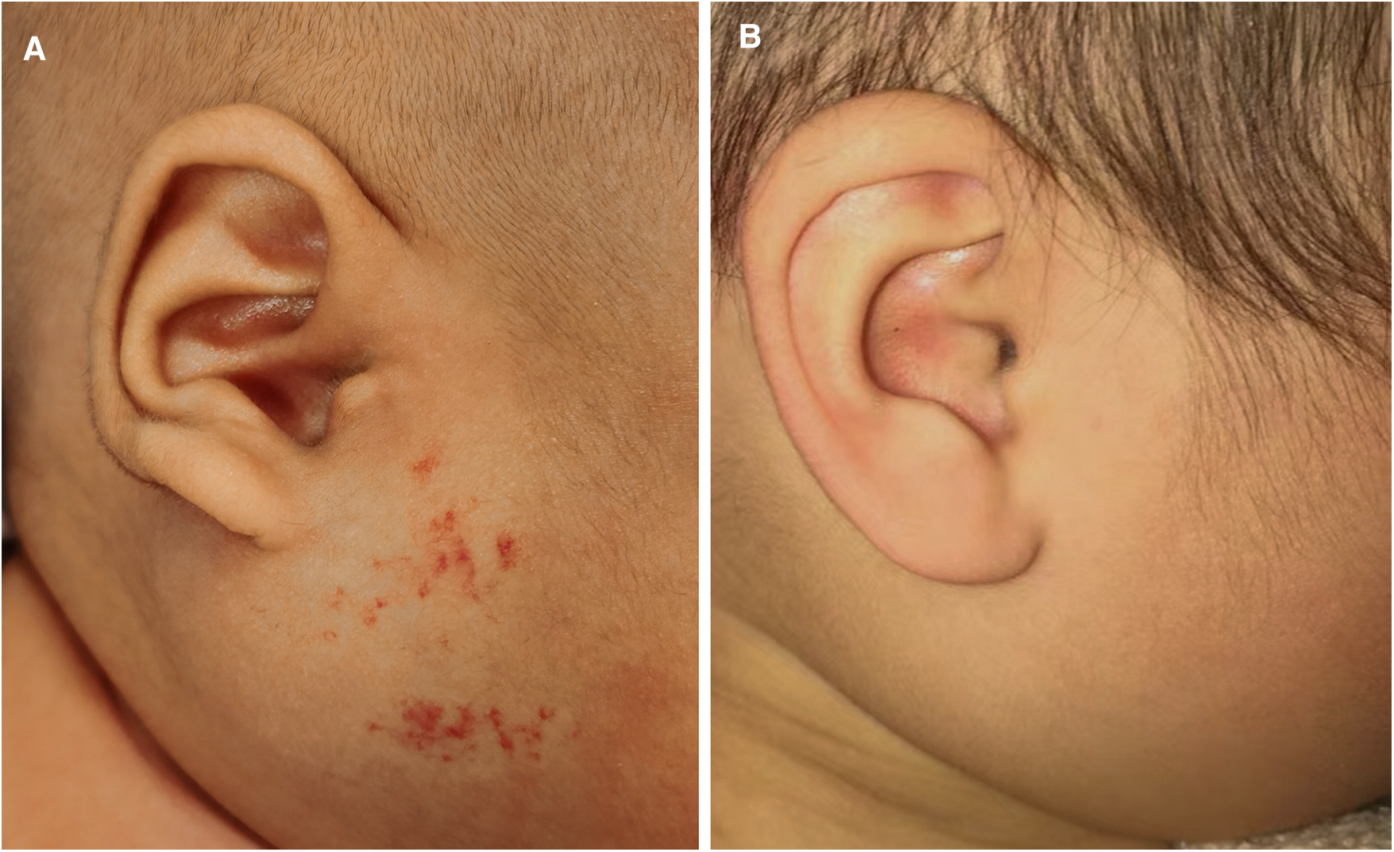
A patient with infantile hemangioma located at face. (**A**) Oral propranolol administration started at 3 weeks of age; (**B**) Oral propranolol administration for 5 months and resulted in excellent final response.

**Figure 2:**
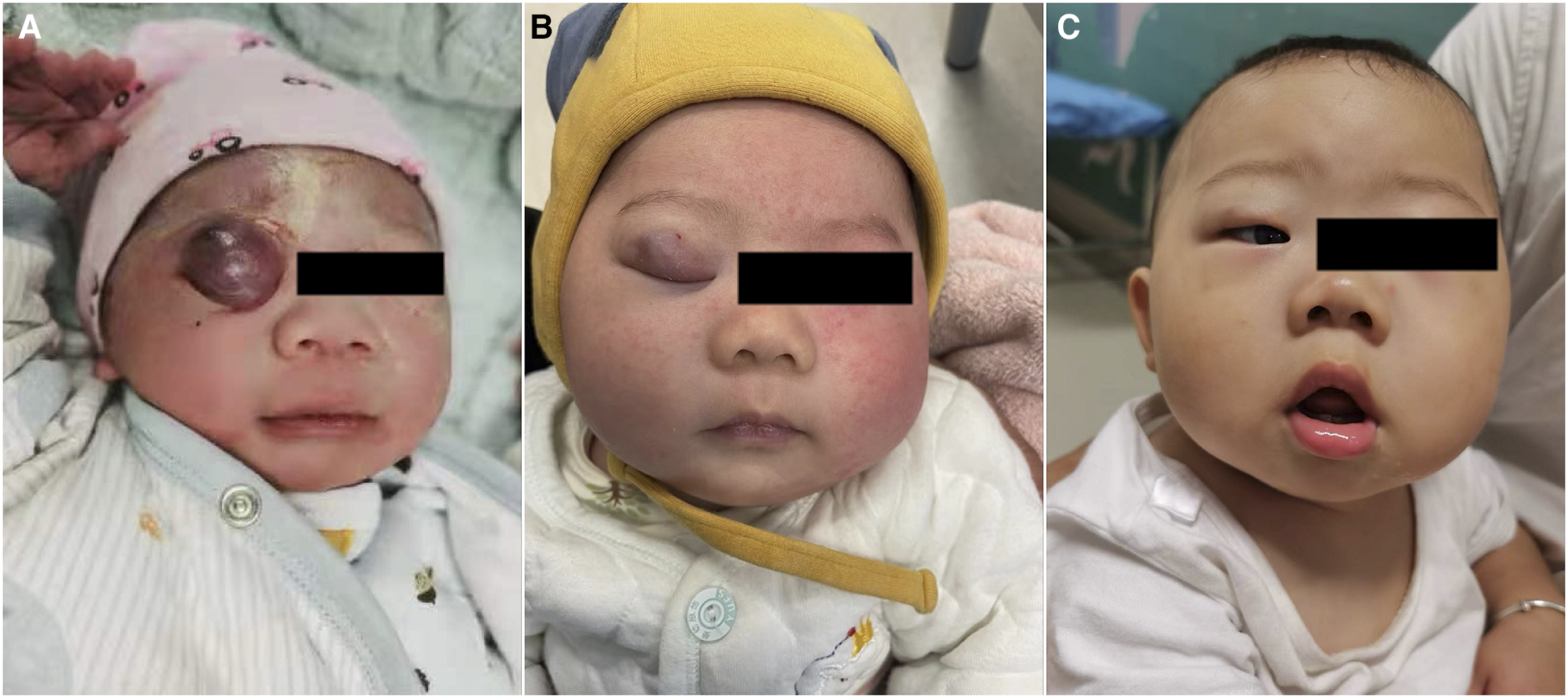
A patient with infantile hemangioma located at upper eyelid. (**A**) Oral propranolol with intralesional injection of lauromacrogol started at 2 weeks; (**B**) oral propranolol and with intralesional injection of lauromacrogol for 3 times in 3 months; (**C**) 6 months after first injection.

**Figure 3:**
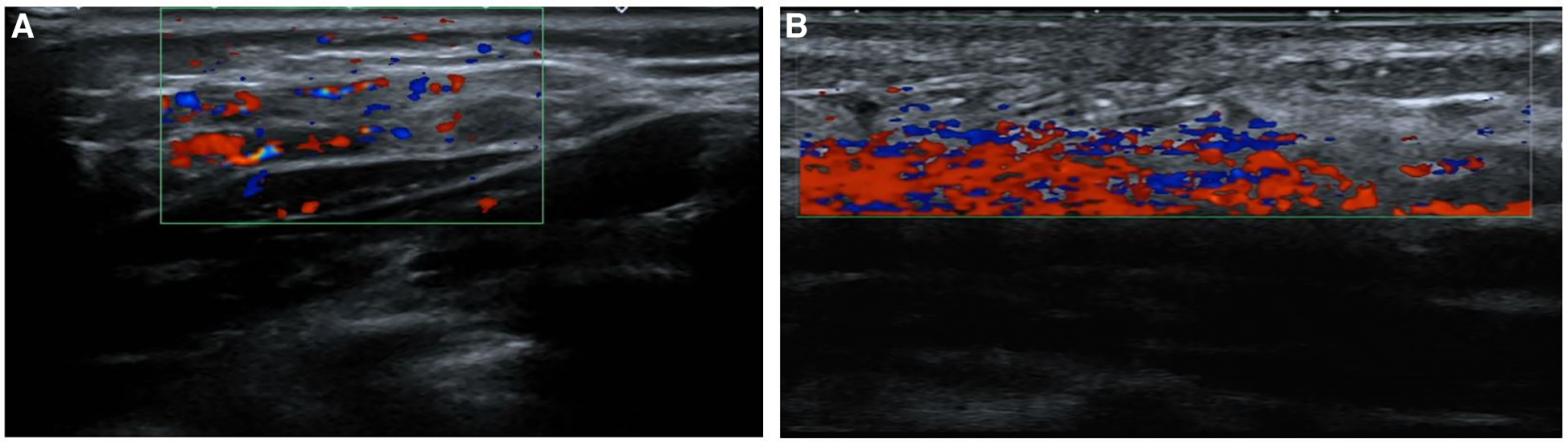
Ultrasound image of a patient with infantile hemangioma located at trunk, and was treated with oral propranolol. (**A**) Before treatment, the tumor body had abundant blood flow signals; (**B**) after 5 months of treatment, the blood flow signal inside the tumor had completely disappeared.

In effective cases of both groups, the initial response was the change of IH color from intense red to lighter red or purple, and the lesion became to be softened. In final response, all patients in both groups showed well improvement in their IH lesions. In PRO group, 11 (37.9%) patients were recorded as grade 1 (excellent final response), 14 (48.3%) patients were recorded as grade 2 (good final response), 4 (13.8%) patients were recorded as grade 3 (fair final response), and no patient was recorded as grade 4 (poor final response). Final response in PRO + LAU group showed 11 (28.9) patients in grade 1, 24 (63.2%) patients in grade 2, 3 (7.9%) patients in grade 3 and no patient in grade 4. No significant differences were found in the final response of both groups. During the process of analysis, we also focused on these patients with excellent final response (Grade 1) in both groups, and compared their treatment time. For these in PRO group, it takes an average of 17.1 ± 5.4 weeks from the start of treatment to cure, and in PRO + LAU group, the average time is 13.7 ± 4.9 weeks, which is shorter, but the *P*-value is 0.24. In addition, no serious side effects (like wheezing, slowed heart rate, hypotension, ulcers, or local infections) were observed in any patient in the PRO group or PRO + LAU group. Final results are shown in Table 2, and the typical cases are shown in Figures 1–3.

**Table 2:** Information of final results of treatment in two groups.

Item	PRO group	PRO + LAU group	*P*-value
Final response, *n* (%)			
Grade 1	11 (37.9%)	11 (28.9%)	0.45
Grade 2	14 (48.3%)	24 (63.2%)	
Grade 3	4 (13.8%)	3 (7.9%)	
Grade 4	0	0	
Treatment duration of patients in Grade 1, weeks (mean, SD)	17.1 ± 5.4	13.7 ± 4.9	0.24

Grade 1 means complete resolution achieved; Grade 2 means with ≥50% reduction in size of infantile hemangioma(IH); Grade 3 means with <50% reduction in size of IH; Grade 4 means no response or worsening of IH.

## Discussion

IH can appear in anywhere of body, like skin, mucous membranes, or underlying viscera, it is a benign proliferation of endothelial cells (14, 15), in our cases, the trunk is the most commonly place where the IH occurred. There were significantly more females than males (≈3:1) in cases and that was in correlation with published data (16).

IH has a slow spontaneous degeneration stage, here a range of complications can develop, which usually lead to pain, disfigure, and dysfunction (4, 17–20). Moreover, half of the IH will have residual tissue after spontaneous regression (21, 22), thus it is necessary for IH to access early interventions to avoid sequelae like telangiectasia, fibrofatty residue or scars.

The pathogenesis of IH is not fully understood. Researchers believe that the hypoxia and renin angiotensin system (RAS) act synergistically as independent pathogenic factors (23–25). Recently, new evidence points out that the RAS is a driving force for the development of IH, and the vasoactive peptide angiotensin II (ATII) is considered as a key regulator of the hemogenic endothelium (26, 27). Moreover, it is similar of the trajectory of serum levels of renin during infancy and childhood to the natural history of IH, and renin levels have also been shown to be physiologically higher in female, that may explain the higher incidence in female group (28). Another research showed IH also can express cathepsins B, D and G and chymase, that means there is an alternative pathway of ATII production, other than the classical RAS, which does not require renin or ACE, can lead to drug resistance to β-blockade and ACE-inhibitors (29).

Propranolol, a nonselective β-blocker, has been studied extensively as an effective systemic therapy for IH, and has been commonly used since 2008 (6, 7, 30). Although efficient, there were still part of IH patients required additional treatment because of significant or serious residual lesions after regression through oral propranolol (8). Intralesional injection of lauromacrogol is a widely confirmed safe way of topical therapy for IH in many medical centers of China, with high efficiency, through destroying the cell membrane, leading to thrombosis, vascular lumen occlusion and irreversible damage to the vascular endothelium (11, 12). On account of the limitation and side effect (like hypoglycaemia, hypotension, bradycardia, bronchospasm, and diarrhea etc.) of single propranolol (31, 32), combination therapy (systemic and topical therapy) was considered to improve the rate of complete regression and reduce the complications of propranolol.

In this study, oral propranolol showed remarkable therapeutic effect, about 86.2% of patients in PRO group received effective treatment with the regression of lesion over 50% within 6 months, and 37.9% of patients in PRO group received complete regression. The treatment effectiveness rate of our patients is similar to that reported in the literature before. However, oral propranolol combined with intralesional injection of lauromacrogol do not show a higher regression rate, although this method also has satisfactory therapeutic effect, it may increase the risk of more side effects related to sclerotherapy in theory. Besides, it is worth to point out that the way combine with intralesional injection of lauromacrogol can shorten the treatment time for these patients with complete regression (Grade 1) although the *P*-value is over 0.05, that means this way may be impactful in shortening the duration of oral treatment only and possible accompanying side effects. Maybe more relevant researches are needed to explore it here.

This is the first study to compare the effectiveness of oral propranolol combined with or without intralesional injection of lauromacrogol in treating IH, and effective suggestion was put forward, which may benefit from avoiding side effects. Meanwhile, our study also has some limits, this is a single center research, and the research period is 6 months, which is too short and make there is a lack of data related to evaluate recurrence of IH.

In conclusion, oral propranolol with intralesional injection of lauromacrogol was a safe treatment strategy for IH in our study, but was not superior to propranolol only in the final cure rate. We still recommend oral propranolol as the first-line treatment option of IH, however, for these patients who cannot tolerate the oral propranolol well, local sclerotherapy can be considered, and combination therapy cannot certainly bring the benefits from shortening duration of oral drug treatment.

## Data Availability

The original contributions presented in the study are included in the article/Supplementary Material, further inquiries can be directed to the corresponding author.
